# Targeting cyclin B1 inhibits proliferation and sensitizes breast cancer cells to taxol

**DOI:** 10.1186/1471-2407-8-391

**Published:** 2008-12-29

**Authors:** Ilija Androic, Andrea Krämer, Ruilan Yan, Franz Rödel, Regine Gätje, Manfred Kaufmann, Klaus Strebhardt, Juping Yuan

**Affiliations:** 1 Department of Obstetrics and Gynecology, School of Medicine, J.W. Goethe-University, Theodor-Stern-Kai 7, 60590 Frankfurt, Germany; 2 Municipal clinics Frankfurt am Main – Hoechst, Gotenstraße 6-8, 65929 Frankfurt, Germany; 3 Department of Medical Microbiology, Immunology and Cell Biology, Cancer Institute, Southern Illinois University, School of Medicine, 911N Rutledge St, Springfield, IL 62702, USA; 4 Department of Radiotherapy and Oncology, School of Medicine, J.W. Goethe-University, Theodor-Stern-Kai 7, 60590 Frankfurt, Germany

## Abstract

**Background:**

Cyclin B1, the regulatory subunit of cyclin-dependent kinase 1 (Cdk1), is essential for the transition from G2 phase to mitosis. Cyclin B1 is very often found to be overexpressed in primary breast and cervical cancer cells as well as in cancer cell lines. Its expression is correlated with the malignancy of gynecological cancers.

**Methods:**

In order to explore cyclin B1 as a potential target for gynecological cancer therapy, we studied the effect of small interfering RNA (siRNA) on different gynecological cancer cell lines by monitoring their proliferation rate, cell cycle profile, protein expression and activity, apoptosis induction and colony formation. Tumor formation *in vivo *was examined using mouse xenograft models.

**Results:**

Downregulation of cyclin B1 inhibited proliferation of several breast and cervical cancer cell lines including MCF-7, BT-474, SK-BR-3, MDA-MB-231 and HeLa. After combining cyclin B1 siRNA with taxol, we observed an increased apoptotic rate accompanied by an enhanced antiproliferative effect in breast cancer cells. Furthermore, control HeLa cells were progressively growing, whereas the tumor growth of HeLa cells pre-treated with cyclin B1 siRNA was strongly inhibited in nude mice, indicating that cyclin B1 is indispensable for tumor growth *in vivo*.

**Conclusion:**

Our data support the notion of cyclin B1 being essential for survival and proliferation of gynecological cancer cells. Concordantly, knockdown of cyclin B1 inhibits proliferation *in vitro *as well as *in vivo*. Moreover, targeting cyclin B1 sensitizes breast cancer cells to taxol, suggesting that specific cyclin B1 targeting is an attractive strategy for the combination with conventionally used agents in gynecological cancer therapy.

## Background

Breast and cervical cancers are the most frequent malignancies in women worldwide [1,2]. Uncontrolled cell proliferation, which is associated with the loss of the proper cell cycle control, is a prominent feature in these cancers. The cell cycle is controlled by a highly conserved family of cyclin-dependent kinases (Cdks) and their regulatory subunits cyclins. Among the cyclins, cyclin B1 plays a pivotal role as a regulatory subunit for Cdk1, which is indispensable for the transition from G2 phase to mitosis. Overexpression of cyclin B1 has been reported in various human tumors, such as breast cancer, cervical cancer, gastric cancer, colorectal cancer, head and neck squamous cell carcinoma and non-small-cell lung cancer [3-9] and its upregulation is closely associated with poor prognosis in various types of cancers including breast cancer [6,10,11]. Moreover, overexpression of cyclin B1 is involved in the resistance to radiotherapy in head and neck squamous cell carcinoma [8] and nuclear cyclin B1-positive breast carcinomas are resistant to adjuvant therapy [11]. More recently, it is reported that both antibodies and T cells are generated in response to aberrant cyclin B1 expression in tumors like breast cancer [12,13], indicating that overexpressed cyclin B1 could serve as one of the signals to initiate the communication between cancer cells and their microenvironment.

The mechanisms accounting for overexpressed cyclin B1 are not yet totally understood. It has been reported that the tumor suppressors p53 and BRCA1 negatively regulate the promoter of cyclin B1 [14-17], whereas the oncogene c-Myc positively regulates the expression of cyclin B1 in cooperation with the loss of p53 [18]. The promoter of cyclin B1 is also upregulated by 17beta-estradiol (E2), insulin-like growth factor I (IGF-I) and prolactin-releasing hormone (PRL), which are considered as the factors contributing to mammary cancer development and progression [19-21]. Moreover, cyclin B1 mRNA is significantly stabilized in cervical cancer cells infected with human papillomavirus type 18 (HPV 18) through upregulating HuR [22], a ubiquitously expressed member of the Hu family of RNA-binding proteins.

The highly expressed cyclin B1, even in G1 phase, binds to its partner Cdk1, which phosphorylates a series of substrates regardless of the cell cycle phase and contributes to the aggressive proliferation in neoplastic tissues [23]. In addition, overexpression of cyclin B1 is related to aneuploidy and high proliferation of human mammary carcinomas [24]. This is consistent with the observation of cyclin B1 overexpression enabling cells to override the G2 DNA damage checkpoint [16,25,26]. Nuclear cyclin B1, together with Cdk1AF, a Cdk1 mutant that cannot be phosphorylated at its inhibitory sites, induced a striking premature mitotic phenotype even after DNA damage [25,26], resulting in accumulation of genomic defects, one hallmark of neoplastic development. More strikingly, enforced expression of cyclin B1 induces tetraploidy, either after mitotic spindle inhibition of nocodazole or in the absence of such inhibition if cyclin B1 is coexpressed with c-Myc [18].

Taken together, deregulation of cyclin B1 is involved in neoplastic transformation and promotes proliferation of tumor cells. Conversely, downregulation of cyclin B1, consequently reducing the activity of Cdk1/cyclin B1, could block the aggressive proliferation of tumor cells. Indeed, our previous data confirm that interfering with cyclin B1 function inhibits proliferation of human tumor cells [27,28]. In the present study, we focus on gynecological cancer cell lines and investigate the effect of small interfering RNA (siRNA) induced cyclin B1 knockdown on tumor cell proliferation. Interestingly, the combination of cyclin B1 siRNA with taxol substantially enhanced the inhibitory effect on proliferation of breast cancer cells. Furthermore, while control HeLa cells were progressively growing, the tumor growth of HeLa cells treated with cyclin B1 siRNA prior to inoculation was strongly inhibited in nude mice, indicating cyclin B1 is indispensable for tumor growth *in vivo*.

## Methods

### Cell culture, reagents and cell synchronization

Cervical cancer cell line HeLa and breast cancer cell lines MCF-7, BT-474, SK-BR-3 and MDA-MB-231 were obtained from DSMZ (Braunschweig). Fetal calf serum (FCS) was purchased from PAA laboratories (Cölbe). Opti-MEM I, oligofectamine, glutamine, penicillin, streptomycin and trypsin were obtained from Invitrogen (Karlsruhe). Taxol was from Mayne Pharma (Haar). Cells were synchronized to G1/S boundary by a double-thymidine block. Briefly, cells were treated with 2 mM thymidine (Sigma-Aldrich, Taufkirchen) for 16 h, released into fresh medium for 8 h and subjected again to thymidine for further 16 h. To obtain prometaphase arrest, after initial thymidine incubation and 8 h release cells were exposed to 50 ng/ml nocodazole (Sigma-Aldrich) for 14 h.

### Transfection of siRNA and the combined treatment with drugs or irradiation

Four siRNAs targeting cyclin B1 (NCBI accession number of cyclin B1: NM 031966) were synthesized by Dharmacon Research, Inc. (Lafayette), referred to as siRNA1-4. siRNA1 against cyclin B1 corresponds to positions 340–360 of the cyclin B1 open reading frame, siRNA2 to positions 476–496, siRNA3 to positions 776–796 and siRNA4 to positions 1302–1322. Control siRNA targeting green fluorescent protein (siGFP) was also purchased from Dharmacon. All siRNAs were 21 nucleotides in length and contained symmetric 3' overhangs of two deoxythymidines.

Cells were transfected with siRNA using transfection reagent oligofectamine, according to the manufacturer's instructions (Invitrogen). In brief, one day prior to transfection, cells were seeded without antibiotics to a density of 50–60%. In all experiments cells were transfected with siRNA1-4 or siGFP at a concentration of 10 nM. Cells were harvested 48 h after siRNA-treatment for cell cycle evaluation, Western blot analysis and kinase assay. The time kinetics of protein expression were carried out at 24 h, 48 h, 72 h and 96 h and proliferation assays were performed at 24 h, 48 h and 72 h after siRNA transfection in MCF-7 cells.

For chemotherapeutic treatment, MCF-7 cells were at first transfected with siRNA and 4 h later followed by treatment of taxol (3 ng/ml). For irradiation, 6 h post transfection cells were exposed to a single dose of 8 Gy at room temperature by a linear accelerator (SL 75/5, Elekta, Crawley, UK) with 6 MEV photons/100 cm focus-surface distance and a dose rate of 4.0 Gy/min. 48 h after transfection of siRNAs cells were harvested for proliferation assay, cell cycle analysis and apoptosis evaluation.

### Western blot analysis and kinase assay *in vitro*

Cell lysis was performed in RIPA buffer (50 mM Tris-HCl pH 8.0, 150 mM NaCl, 1% NP-40, 0.5% Na-desoxycholate, 0.1% SDS, 1 mM Na_3_VO_4_, 1 mM phenylmethylsulphonyl-fluoride (PMSF), 1 mM Dithiothreitol (DTT), 1 mM NaF, and protease inhibitor cocktail Complete (Roche, Mannheim)). Total protein was separated by using 12% sodium dodecyl sulfate-polyacrylamide gel electrophoresis (SDS-PAGE) and then transferred to Immobilon-P membranes (Millipore, Bedford, MA). Membranes were exposed to corresponding antibodies for 1 h in PBS containing 5% slim milk, washed with phosphate-buffered saline (PBS) containing 0.2% Tween-20, incubated subsequently with secondary antibodies for 1 h. Finally, the protein bands were visualized with the enhanced chemiluminescence reagent (ECL, Pierce, Rockford). Mouse monoclonal antibodies against cyclin B1 (1:5,000), Cdk1 (1:2,000), anti-mouse secondary antibodies (1:4,000) and anti-rabbit secondary antibodies (1:4,000) were purchased from Santa Cruz (Heidelberg). Rabbit polyclonal antibodies against PARP (poly(ADP-ribose) polymerase, 1: 1000) were from Cell Signaling Technology (Beverly). Mouse monoclonal antibodies against β-actin (1:200,000) were obtained from Sigma-Aldrich. Western blots were quantified by applying a Kodak gel documentation system (model 1D 3.5) and standardized with loading control. For kinase assays *in vitro*, antibodies against cyclin B1 (Santa Cruz, Heidelberg) were used for immunoprecipitation from 600 μg of cellular extracts. 0.5 μg histone H1 (Calbiochem, Darmstadt) served as substrate for each reaction. Kinase assays were performed as previously described [29].

### Cell proliferation, cell cycle analysis and apoptosis assay

Cell viability was assessed by trypan blue staining. The proliferation rate of cells was determined at indicated time points by counting cell numbers with a hemacytometer. All experiments were performed in triplicate. For cell cycle analysis, cells were harvested, washed with PBS and fixed in 70% chilled ethanol at 4°C for 30 min, then treated with 1 mg/ml of RNase A (Sigma-Aldrich) and stained with 100 μg/ml of propidium iodide for 30 min. The DNA content of 10,000 cells was determined with a fluorescent-activated cell sorter FACScan (Becton Dickinson Biosciences, Heidelberg). The data were analysed with cell cycle analysis software ModFit LT 2.0 (Verity Software House, Topsham, ME). Most of the experiments were performed in triplicate. Indirect immunofluorescence staining for subcellular cyclin B1 localization and DNA were carried out as previously described [29]. Apoptosis was assessed using Vybrant™ apoptosis assay kit according to the manufacturer's instructions (Molecular Probes, Leiden).

### Colony formation assay and *in vivo *experiments

MCF-7 cells were treated with siRNA1-3 or siGFP for 48 h and harvested for colony formation assays. Briefly, cells were seeded in 24 well-plates at a density of 200 cells/well into culture medium containing 0.3% agar (Roth, Karlsruhe) overlaying 0.5% agar. Cells were cultured at 37°C with 5% CO_2_, and colonies were counted 4 weeks later using a microscope (Zeiss, Oberkochen). The colony number in control sample was referred as 100% by quantification.

As to experiments *in vivo*, HeLa cells were treated with siRNA3 or siGFP and harvested after 48 h. HeLa cells (1 × 10^6^) were resuspended in 300 μl of 0.9% NaCl and subcutaneously injected into both flanks of nude mice. Each group contained 4 mice. Three weeks after inoculation the tumor sizes were measured every 3–4 days using callipers and the tumor volumes were calculated according to a standard formula: π/6 × length × width^2^. The tumor volumes within the group were represented by the mean value. All mice were properly treated in accordance with the guidelines of the local animal committee.

### Statistic analysis

For assays *in vitro*, Student's *t*-tests were used to evaluate the significance of difference between control cells and siRNAs-treated cells. Differences were considered as statistically significant when *p *< 0.05. With xenograft mouse model, the significant difference between the siGFP-treated group and siRNA3-treated group was analyzed by Mann-Whitney U test.

## Results

Cancer cell lines lend themselves as useful models to further our understanding of gynecological cancers such as breast and cervical cancer. To study the function of cyclin B1 in breast cancer cells we selected cancer cell lines MCF-7, BT-474, SK-BR-3 and MDA-MB-231, as they represent the best characterized cell lines for breast cancer research. While MCF-7 and BT-474 express both estrogen receptor (ER) and progesterone receptor (PgR), SK-BR-3 and MDA-MB-231 cells lack those receptors [30]. Unlike SK-BR-3 and BT-474, which are Her-2/*neu*-positive, MCF-7 cells only exhibit a basal level of Her-2/*neu *[30]. In addition, MCF-7 cells express high amounts of markers typical of the luminal epithelia phenotype of breast cells, whereas BT-474 and SK-BR-3 cells exhibit a weakly luminal epithelia-like phenotype. Distinct from the two phenotypes above, MDA-MB-231 represents a highly invasive “mesenchymal-like“ breast cancer cell line by expressing a high level of vimentin. In addition, the cervical cancer cell line HeLa was selected, because it represents the most extensively studied cancer cell line thus far.

### Specific downregulation of cyclin B1 with siRNA

**Figure 1:**
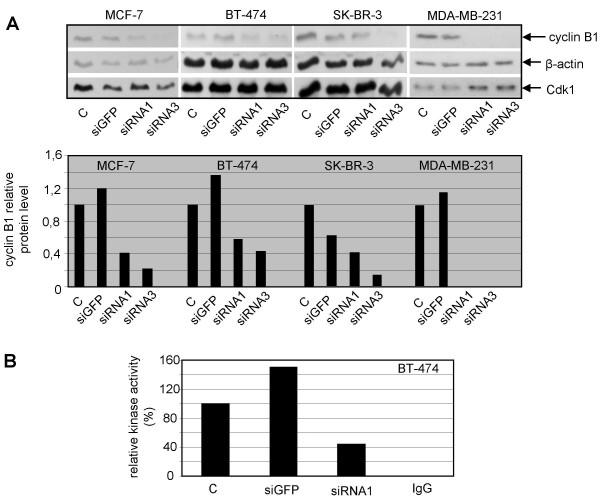
**Reduced cyclin B1 protein and kinase activity of Cdk1/cyclin B1**. A. Upper panel: Breast cancer cells MCF-7, BT-474, SK-BR-3 and MDA-MB-231 cells were treated with cyclin B1 siRNA1, siRNA3, GFP siRNA (GFP) or without treatment (C) for 48 h. Cells were harvested and cellular lysates were prepared for Western blot analyses with antibodies targeting cylin B1, Cdk1 and β-actin. The later served as loading control. Lower panel: Quantification of cyclin B1 levels from Western blots (upper panel), normalized to β-actin. B. Kinase assay of Cdk1/cyclin B1 *in vitro*. BT-474 cells were treated with cyclin B1 siRNA1 or siGFP or without treatment (C). 24 h later cells were lysed and Cdk1/cyclin B1 complex was immunoprecipitated by using antibodies against cyclin B1 from cellular extracts. Normal IgG served as negative control. Kinase assays were carried out with the precipitates in the presence of histone H1 as substrate.

We were at first interested whether breast cancer cell lines are similarly sensitive to specific downregulation of cyclin B1 by siRNA. As shown in Fig. 1A, while the protein level of cyclin B1 in MDA-MB-231 was almost undetectable after treatment with siRNA1 or siRNA3, 33% and 16% of cyclin B1 were still detectable in MCF-7 cells after treatment with siRNA1 and siRNA3, respectively, relative to the protein level of cyclin B1 in control cells. In contrast to cyclin B1, β-actin was not affected. The protein level of Cdk1, the catalytic partner of cyclin B1, hardly changed, indicating cyclin B1 knockdown by siRNAs was specific. Treatment of BT-474 and SK-BR-3 cells with siRNA1 and siRNA3 also reduced cyclin B1 levels, albeit to a lower extent as compared to MDA-MB-231 and MCF-7 cells. Downregulation of cyclin B1 was also corroborated by indirect immunofluorenscence staining with monoclonal specific antibodies against cyclin B1 (data not shown). Furthermore, in consistence with downregulation of cyclin B1 protein, the kinase activity of Cdk1/cyclin B1 was decreased to 28% in cellular extracts from siRNA1-treated BT-474 cells as compared to siGFP-treated cells (Fig. 1B). Similar results were also obtained in MCF-7 cells after siRNA administration (data not shown).

### Proliferation is inhibited in breast cancer cells with reduced cyclin B1

**Figure 2:**
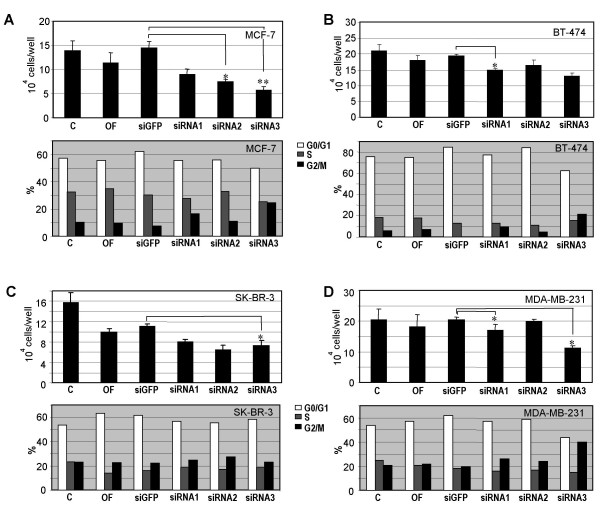
**Inhibited proliferation after downregulation of cyclin B1**. MCF-7 (A), BT-474 (B), SK-BR-3 (C) and MDA-MB-231 cells (D) were treated with siRNA1-3, or siGFP or oligofectamine alone (OF). After 48 h cells were harvested for cell number counting (upper panels in Fig. 3 A-D) and for cell cycle analysis (lower panels in Fig. 3 A-D). Cells without any treatment (C) served as control. The results of cell numbers are expressed as mean ± SD (*n *= 3) and statistically analysed. *P < 0.05, **P < 0.01.

As Cdk1/cyclin B1 is essential for the initiation of mitosis and required for cell division we subsequently studied the impact of cyclin B1 downregulation on cell proliferation rate. Expectedly, a reduced proliferation was observed in all four breast cancer cell lines after 48 h treatment with siRNAs as compared to control cells treated with siGFP (Fig. 2). In particular, MCF-7 cells exhibited a strong inhibition of proliferation, followed by MDA-MB-231, BT-474 and SK-BR-3 cells after 48 h siRNA1-3 treatment against cyclin B1 (Fig. 2A–D, upper panels). Analyses of cell cycle distribution displayed an accumulation of cells in G2/M phase, suggestive of a G2/M arrest, after treatment with siRNA1-3 targeting cyclin B1 (Fig. 2A–D, lower panels).

### Time kinetics of siRNA treatment in MCF-7 cells

**Figure 3:**
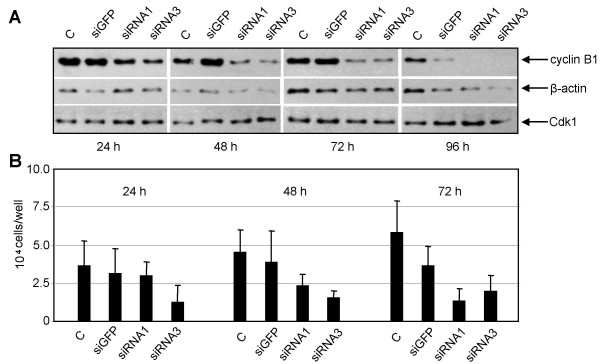
**Time kinetics in MCF-7 cells**. MCF-7 cells were treated with cyclin B1 siRNA1, siRNA3, siGFP or without treatment (C). Treated cells were harvested at different time points as indicated for Western blot analyses with antibodies against cyclin B1, Cdk1 and β-actin (A) and for cell number determinations (B). The results of cell numbers are expressed as mean ± SD (*n *= 3).

In order to study the effect of cycin B1 knockdown on proliferation, we subjected MCF-7 cells to more detailed time dependent analysis. MCF-7 cells were treated with siRNA1 or siRNA3 and harvested for Western blot analysis and proliferation assay at the indicated time points. The reduction of cyclin B1 protein levels were evident 24 h after siRNA1/3 treatment as compared to control cells (Fig. 3A). This effect increased dramatically with time and after 96 h cyclin B1 protein became undetectable. In line with the reduction of cyclin B1, the proliferation rate of MCF-7 cells was clearly inhibited at 24 h with siRNA3 treatment and the inhibition became more striking with longer exposure to siRNA1 (Fig. 3B).

### Suppression of cyclin B1 renders cells more susceptible to taxol

**Figure 4:**
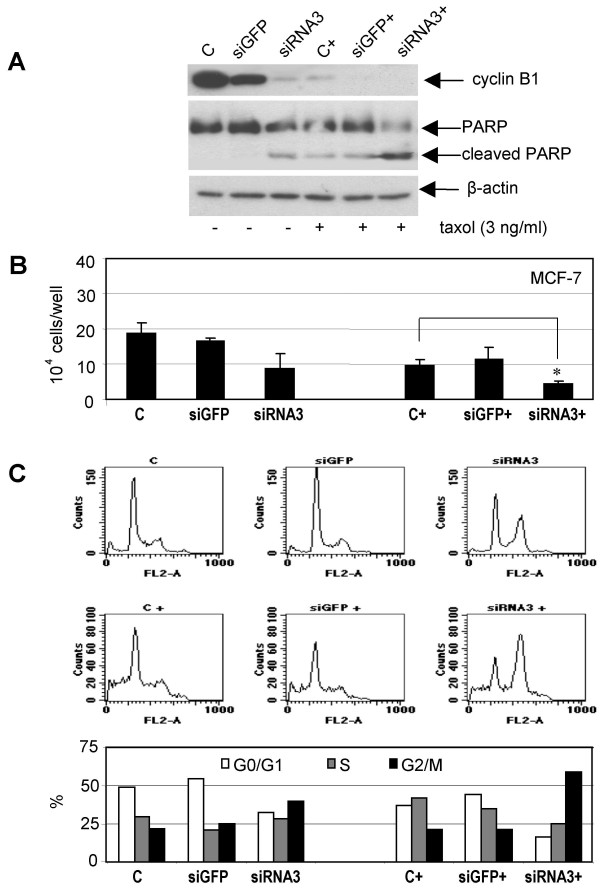
**More sensitive to taxol in MCF-7 cells with reduced cyclin B1**. A. MCF-7 cells were transfected with siRNA3 or siGFP and 4 h later followed with treatment of taxol (C+, siGFP+ and siRNA3+) or without taxol (C, siGFP and siRNA3). 48 h after transfection cells were harvested for Western blot analyses with antibodies as indicated. β-actin served as loading control. PARP: poly (ADP-ribose) polymerase. B. Cells were treated as in A and cell numbers were counted. The results are expressed as mean ± SD (*n *= 3) and statistically analysed. *P < 0.05. C. MCF-7 cells were treated as in A and cell cycle was analyzed (upper panel) and distribution of cell cycle population was quantified (lower panel).

Taxol (Paclitaxel^®^), a taxane frequently used in multidrug regiments for the therapy of several solid tumors, binds to the β-subunit of tubulin, thereby impairing the dynamics of microtubules by promoting their polymerization, leading to mitotic arrest and apoptosis [31]. In order to explore cyclin B1 knockdown as a possible combination with taxol, we transfected MCF-7 cells with siRNA3 and followed by further incubation with taxol. Cells were harvested 48 h posttransfection. As depicted in Fig. 4A, cyclin B1 protein level was strongly reduced after siRNA3 treatment. Cyclin B1 level was also decreased after treatment with taxol at a low dosage of 3 ng/ml, possibly due to the induction of apoptosis, and almost disappeared when cells were pre-treated with siRNA3 (Fig. 4A, upper panel). Indeed, PARP (poly(ADP-ribose) polymerase) was cleaved in cells treated with taxol and more strongly cleaved when siRNA3 was used together with taxol (Fig. 4A, middle panel), indicating that the combined treatment triggers more robust apoptotic response. In addition, proliferation was inhibited to a higher extent after the combined treatment (Fig. 4B). The results were further correlated with the cell cycle analysis: a G2/M population was more prominent in MCF-7 cells after the combined treatment, compared to cells exposed to siRNA3 alone (Fig. 4C). The data indicate that the combined therapy activates more strongly caspase-3 independent apoptotic pathways in MCF-7 cells as the *caspase-3 *gene is deleted in MCF-7 cells [32]. Similar results were also obtained in MDA-MB-231 cells: while siRNA3 or taxol alone reduced proliferation by 33% and 31%, respectively, relative to siGFP treatment, the combination of siRNA3 with taxol resulted in a 65% reduction of proliferation (data not shown). Thus, the combined action of cyclin B1 knockdown together with taxol enhances antiproliferative and proapoptotic responses in breast cancer cells. Furthermore, we investigated the combination of knockdown of cyclin B1 with irradiation. Irradiation enhanced the G2/M population and induced more apoptosis in cyclin B1 reduced MCF-7 cells, compared to control cells (data not shown).

### Impaired colony-forming ability and inhibited tumor growth

**Figure 5:**
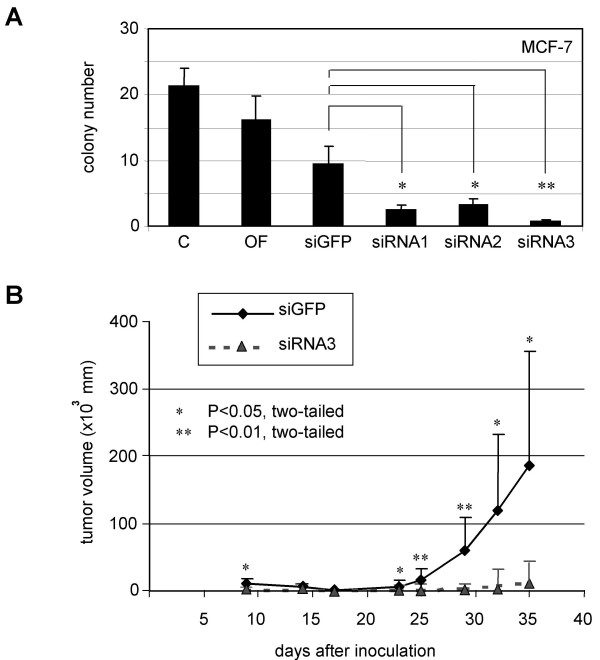
**Impaired colony-forming ability and reduced tumor growth**. A. Colony formation assay. MCF-7 cells were treated with siRNA1-3 or siGFP. 48 h after transfection cells were transferred to soft-agar plates for further incubation. 4 weeks later the colony numbers were scored with a microscope. The colony number of control cells was regarded as 100% for calculation. The data were expressed as mean ± SD (*n *= 3) and analysed by Student's *t*-test. *P < 0.05, **P < 0.01. B. Xenograft experiment in nude mice. 1 × 10^6 ^of HeLa cells treated with siRNA3 or control siGFP were subcutaneously injected into each flank of nude mice. Each group contained 4 mice. Tumor sizes were measured every 2–3 days and tumor volumes were calculated. The data were statistically analysed by Mann-Whitney U test. *P < 0.05, **P < 0.01.

Anchorage independent cell growth is one of the hallmarks of malignant tumor cells. Given the notion of cyclin B1 deregulation being involved in neoplastic transformation and associated with malignancy grade of tumors, we wondered whether knockdown of cyclin B1 protein might translate into reduced colony-forming ability in MCF-7 cells. As shown in Fig. 5A, the colony numbers of MCF-7 cells treated with siRNA1-3 were strongly reduced, in particular, in MCF-7 cells treated with siRNA3, compared to controls.

Cervical carcinoma HeLa cells express a high level of cyclin B1 (data not shown). In cell culture, only 25–30% HeLa cells were left after 48 h treatment with siRNA3 targeting cyclin B1 (data not shown). To further address whether cyclin B1 is required for aggressive growth of tumors *in vivo*, a xenograft experiment with HeLa cells was performed in nude mice. Mice were inoculated with HeLa cells treated 48 h with siRNA3 targeting cyclin B1 or siGFP. As shown in Fig. 5B, tumor growth of HeLa cells treated with siRNA3 prior to inoculation was effectually retarded, in comparison with the growth of control HeLa tumors. The data suggest that cyclin B1 is indeed required *in vivo *for promoting proliferation of tumor cells and the reduction of cyclin B1 slows down the tumor growth.

## Discussion

The prognosis of breast and cervical cancer patients has been improved during recent years, related partly to sophisticated surgery, radiotherapy and adjuvant systemic therapy. Despite these advances, these cancers remain major clinical problems by causing considerable morbidity and mortality in women worldwide. Apart from the standard approaches, novel potent molecular agents for anticancer therapy are in great demand.

In this communication we show that the knockdown of cyclin B1, the regulatory subunit of Cdk1, inhibited cell proliferation and induced apoptosis in various breast and cervical cancer cell lines. Importantly, siRNA mediated cyclin B1 knockdown in combination with chemotherapeutical agent taxol, enhanced the antiproliferative effect on breast cancer cells. Interestingly, the reduction of cyclin B1 in MCF-7 cells impaired colony-forming ability, a hallmark of malignancy in tumor cells. Moreover, while control HeLa cells were progressively growing, the tumor growth of HeLa cells treated with siRNA targeting cyclin B1 prior to inoculation was strongly inhibited in nude mice, indicating cyclin B1 is indispensable for tumor growth *in vivo*. Taken together, the data strengthen the notion of cyclin B1 being required for the survival and proliferation of breast and cervical cancer cells and depletion/downregulation of cyclin B1 inhibits proliferation of cancer cells *in vitro *as well as *in vivo*.

Recent genetic evidence demonstrates that Cdk1 is the only Cdk sufficient to drive the mammalian cell cycle because embryos from Cdk1^-^/^- ^mice fail to develop to the morula and blastocyst stages, whereas mouse embryos lacking all interphase Cdks (Cdk2, Cdk3, Cdk4 and Cdk6) undergo organogenesis and develop to midgestation [33]. These data underscore that Cdk1 is essential for cell cycle regulation and a major force driving cell proliferation. Cyclin B1, the regulatory subunit of Cdk1, controls the activity of Cdk1 as it associates with and thereby activates Cdk1, regulates its nuclear translocation and passively mediates its inactivation when cyclin B1 is degraded at anaphase transition. Cyclin B1 is fundamental for cell proliferation. Uncontrolled expression of cyclin B1 is associated with neoplastic transformation and gynecological cancer development [5,9,11,34,35]. Overexpression of cyclin B1 is believed to confer therapy resistance [8,11]. Thus, targeting cyclin B1, leading consequently to the inactivation of Cdk1, could be a promising specific strategy for cell cycle intervention against breast and cervical cancer.

In this work, as a proof-of-concept, the RNA interference was used to downregulate/deplete cyclin B1 and a clear antiproliferative effect was observed in all cancer cell lines studied. Among the breast cancer cell lines investigated, MCF-7 cells exhibited the strongest inhibitory effect on cell proliferation after cyclin B1 siRNA treatment, followed by MDA-MB-231, SK-BR-3 and BT-474 cells (Fig. 2), which possibly correlates with the cyclin B1 level in exponential growing status of each cell line (data not shown). Although the protein level of cyclin B1 in MDA-MB-231 cells was nearly undetectable after siRNA1 or siRNA3 transfection, the inhibitory impact was moderate (Fig. 2D), suggesting the proliferation of MDA-MB-231 cells is not necessarily dependent on the normal level of cyclin B1 and the little amount of remaining cyclin B1 might be sufficient for the survival of MDA-MB-231 cells. Finally, SK-BR-3 and BT-474 cells were also not as sensitive to siRNA treatment (Fig. 2B and 2C) as HeLa or MCF-7 cells. This could be due to the cellular context of SK-BR-3 and BT-474 cells, e.g. Her-2/*neu*+, which very often leads to a hormone-independent proliferation of cells. Thus, unlike in MCF-7 cells, targeting Her-2/*neu *or other factors promoting G1/S transition could be more effective for inhibiting cell cycle progression in SK-BR-3 and BT-474 cells, which has been shown by our previous study [36]. On that account, specific targeting of oncogene(s) in individual cancer cell lines, like Her-2/*neu *in SK-BR-3 and BT-474 cells, or cyclin B1 in MCF-7, could improve breast cancer therapy. Collectively, downregulation/depletion of cyclin B1 worked effectively in all gynecological cancer cell lines tested. However, only in some cell lines, such as MCF-7 and HeLa, cyclin B1 knockdown resulted in a strong proliferative inhibition, most likely because proliferation in those cell lines is more dependent on high cyclin B1 levels as compared to other cell lines.

Taxane drugs represent the most important class of anticancer agents and are integrated in multidrug-regiments for the therapy of several solid tumors including gynecological cancers. Despite their relevant contribution in ameliorating the quality of life and overall survival of cancer patients, drug resistance and site-effects hamper its wide usage. Therefore, it is desirable to find new ways of lowering drug dosage without losing effectiveness to limit side-effects and possibly also to slow down drug resistance. In this work, cyclin B1 siRNA in combination with taxol, blocking entry into mitosis and targeting the transition of metaphase to anaphase, respectively, demonstrated a high efficacy in inhibiting proliferation of MCF-7 cells. The data suggest that specific targeting of cyclin B1 could sensitize some gynecological cancer cells, like MCF-7 and MDA-MB-231 cells, to conventional chemotherapeutic agents like taxol, thereby reducing their side-effects by lowering their dosage.

Taken together, the data from this work further strengthen the notion that cyclin B1 could be an attractive target for potential anticancer therapy. Inhibiting cyclin B1 function in combination with chemotherapeutic drugs could reinforce the antiproliferative effect in a subset of cancers. As RNA interference still faces the major challenge of systematic delivery [37], an alternative strategy could be small molecule inhibitors targeting cyclin B, as its crystal structure is recently published [38]. In parallel to Cdk inhibitors, which have been extensively under clinical investigations, small molecule inhibitors against cyclin B1 could open up a new door for specific molecular cancer therapy by interfering with its protein stability, binding capacity to Cdk1 or its subcellular localization.

## Conclusion

This work demonstrates that cyclin B1 is required for survival and proliferation of breast and cervical cancer cells. Downregulation of cyclin B1 inhibits proliferation of tumor cells *in vitro *as well as *in vivo *and sensitizes breast cancer cells to taxol. The data indicate that the combination of reducing cyclin B1 with chemotherapeutic drugs could be a new strategy for molecular intervention in a subset of breast cancers.

## Competing interests

The authors declare that they have no competing interests.

## Authors' contributions

IA and AK conducted cell cycle analyses, proliferation assays, Western blot analyses, apoptosis assays and mouse xenograft experiments *in vivo*. RY performed the kinetics of MCF-7 cells and soft-agar assays. FR is involved in the combination therapy and assays. MK and RG coordinated this project. KS co-supervised this study and supported the manuscript writing. JY designed and supervised this study, and drafted the manuscript. All the authors read and approved the final manuscript.

## Pre-publication history

The pre-publication history for this paper can be accessed here:


